# Associations of five blood heavy metals with hepatitis B virus infection and immunity in adults: a cross-sectional study

**DOI:** 10.1186/s12889-024-17799-1

**Published:** 2024-01-23

**Authors:** Xinpeng Li, Lei Bi, Lu Han

**Affiliations:** Department of Clinical Laboratory, Public Health Clinical Center of Chengdu, Chengdu, 610000 China

**Keywords:** Hepatitis B virus, Heavy metals, Lead, NHANES

## Abstract

**Background:**

Heavy metal pollution has emerged as a significant concern for human health, prompting increased awareness of its potential adverse effects. While previous research has established a connection between heavy metals and liver function biomarkers, the specific relationship between heavy metals and HBV infection remains unexplored. This cross-sectional study aims to investigate the potential correlations between five blood heavy metals - lead, cadmium, mercury, manganese, and selenium - and the presence of HBsAg, HBsAb, and HBcAb in adults.

**Methods:**

The study utilized data from NHANES 2007–2018. Participants were classified into four groups based on their infectious status, and the association between heavy metals and HBV infection was analyzed using multiple logistic regression and stratification analysis.

**Results:**

A total of 8431 participants were included, with 5 436 classified as Susceptible, 1 765 as Vaccinated, 865 as Natural Infection, and 103 as Acute/Chronic HBV Infection. The Vaccinated group exhibited a lower mean age (34.52 ± 14.16 years) compared to the other groups. Statistically significant differences in heavy metal concentrations (except selenium) were observed among the groups (*P* < 0.001). After adjusting for covariates, lead was significantly associated with HBV infection (Q2: OR 2.37, 95%CI 1.04–5.39; Q3: OR 2.34, 95%CI 1.01–5.40), and positive trends were observed for high blood concentrations of mercury (Q4: OR 3.03, 95%CI 1.31–7.04) and manganese (Q4: OR 2.52, 95%CI 1.20–5.28). Furtherly, the presence of lead reduced the protection of HBsAb (Q2: OR 0.84, 95%CI 0.73–0.97; Q3: OR 0.77, 95%CI 0.66–0.90; Q4: OR 0.83, 95%CI 0.70–0.98). Subgroup analysis indicated that cadmium was associated with an increased risk of HBV infection in Asians (OR 1.36, 95%CI 1.03–1.78) and individuals with a BMI range of 25 to 30 (OR 1.60, 95%CI 1.17–2.18).

**Conclusions:**

The study’s findings suggest a correlation between elevated blood Pb concentrations and reduced immunization rates against hepatitis B. Individuals with a positive HBsAg exhibit lower blood Se concentrations and higher blood Hg and Mn concentrations.

## Introduction

Hepatitis B is an acute or chronic infectious disease caused by the enveloped DNA virus - hepatitis B virus (HBV) [1]. HBV is primarily spread through percutaneous or mucosal exposure to infected blood and various body fluids, mother-to-child transmission, and sexual contact [2]. HBV infection affects around 296 million individuals globally, with a disproportionate impact on sub-Saharan Africa and East Asia [3]. Dane particles are complete and infectious HBV virions, consisting of an outer envelope containing HBV surface antigen (HBsAg), Pre S1, and Pre S2, while the core surface is composed of HBV core antigen (HBcAg) [4]. Hepatitis B surface antibodies, abbreviated as HBsAb, are protective neutralizing antibodies generated after exposure to HBsAg or through vaccination [5]. HBcAb, or hepatitis B core antibodies, are immunoglobulins produced by the body in response to the presence of HBcAg. These antibodies serve as serological markers to indicate previous or ongoing HBV infection, as they can be detected during both acute and chronic phases of the disease [6]. Acute hepatitis B is generally a self-limited condition characterized by hepatocellular necrosis and inflammation. Chronic hepatitis B (CHB) poses a significant public health concern, characterized by the continued presence of HBsAg for six months or more. The major complications of CHB include cirrhosis and hepatocellular carcinoma (HCC) [1].

Heavy metals are metallic elements that possess a relatively high density (> 5 g/cm3) and atomic weight compared to water, widely distributed in the Earth’s crust, rocks, soils, ores, and minerals [8]. Some heavy metals play essential roles in biological processes, including oxygen transportation, cellular growth, metabolism, and glucose utilization [9]. The emissions of heavy metals from various industrial activities have led to excessive pollution of water, soil, flora, fauna, and air [10]. Human beings can be exposed to large quantities of heavy metals acutely through inhalation, ingestion, or dermal contact, or experience chronic exposure to low levels of heavy metals over an extended period [11]. Workers in waste management facilities, welders, and individuals employed in various occupations face potential risks of respiratory or skin exposure to heavy metals, along with their associated toxic and hazardous effects [12]. Cadmium (Cd) may cause proximal tubular cell damage and increase the risk of osteoporosis and lung cancer [14]. The heavy metals lead (Pb), mercury (Hg), and manganese (Mn) are neurotoxins. Pb has been linked to increased blood pressure in adults [15], Hg is associated with fetal microcephaly [16], and Mn can cause respiratory tract irritation, leading to coughing and asthma. Furthermore, Mn can have detrimental effects on organs such as the liver, kidneys, and cardiovascular system [17]. Selenium (Se) plays essential biological roles in the human body, such as participating in antioxidant reactions, supporting immune system function, and contributing to thyroid hormone metabolism. However, in cases of Se poisoning, it can lead to damage in the gastrointestinal system, abnormal skin and nails, and disruption of thyroid function [18]. Additionally, a study reveals that Pb and Cd emerge as the most significant metals that accumulate in the bloodstream of cigarette smokers [19].

An increasing body of evidence links heavy metal exposure to various human chronic diseases [9]. Pb, Cd, Se, and methyl Hg have been found to have varying degrees of impact on cognitive function. Cd and Pb were negatively associated with CERAD immediate recall scores, while Se showed a strong positive association [20]. Constant exposure to a combination of heavy metals has been associated with obesity and its related chronic conditions, including hypertension and type 2 diabetes [21]. Moreover, heavy metal exposure has been found to be linked to various liver disorders as well [22]. Liver function parameters, including alanine transaminase (ALT), aspartate transaminase (AST), gamma-glutamyl transferase (GGT), alkaline phosphatase (ALP), and the ALT/AST ratio, showed significant associations with the concentration of blood heavy metals (Hg, Mn, Pb, Cd) [24]. In the non-alcoholic fatty liver disease (NAFLD) population, a study demonstrated that a urinary mixture of fourteen metals positively correlated with Ln CAP in both the BKMR and qgcomp models. This finding supports the notion of the effects of the heavy metal mixture on NAFLD [25]. Research studies have also reported a correlation between heavy metals and viral hepatitis. A study investigating the immune response to the hepatitis B vaccine in children with chronic Pb exposure revealed that nearly 50% of chronically exposed children did not develop sufficient immunity to hepatitis after vaccination. This suggests that the immune response to the hepatitis B vaccine and immune system suppression may pose potential risks to children who were chronically exposed to Pb [26]. However, there is still little study on heavy metal exposure and hepatitis B infection and immunity, especially except for other heavy metals commonly found in blood such as lead and cadmium. Therefore, the objective of this study was to explore the relationship between heavy metals and HBV markers using the National Health and Nutrition Examination Survey database (NHANES).

## Methods

### Data source and study population

**Fig. 1:**
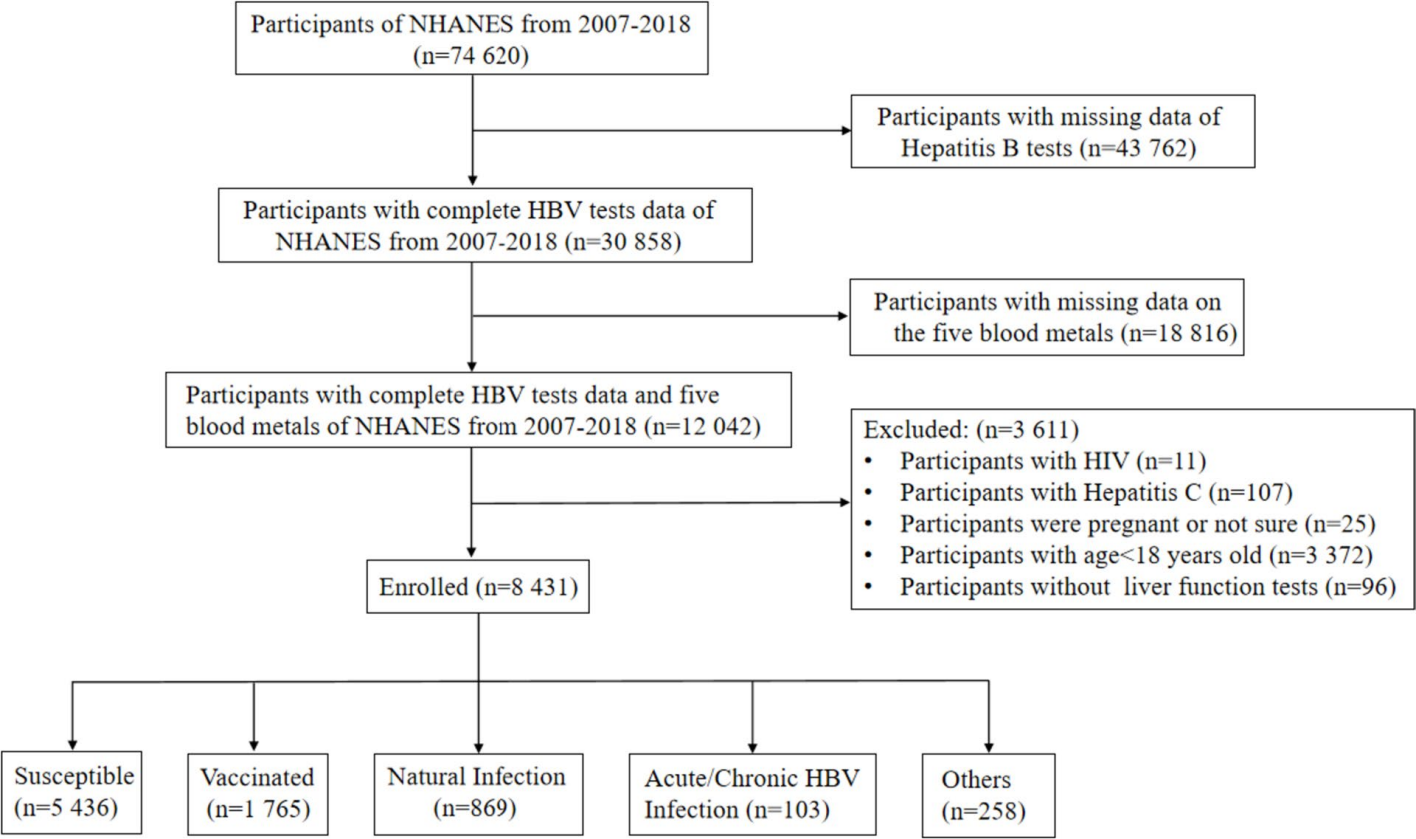
Flow chat of the participants selection

All data were derived from the NHANES database (https://www.cdc.gov/nchs/nhanes/). NHANES is a program of study designed to assess the health and nutritional status of adults and children in the United States. From a total of 74 620 participants, we initially excluded 43 762 individuals due to the lack of Hepatitis B test data. Subsequently, 18 816 individuals with missing data on the five blood metals were further excluded. Finally, we removed 11 participants with HIV, 25 participants with uncertain pregnancy status, 3 372 participants under the age of 18, and 96 participants without liver function test results. This resulted in a final sample size of 8 431 participants, which was used for the subsequent group-based analysis (Fig. 1).

### Measurements of heavy metals

The detection method for heavy metals is based on the blood multi-element analysis by ICP-DRC-MS, as referenced in NHANES. A small amount of whole blood anticoagulant specimen is extracted from patients and vortex-mixed to ensure an accurate reflection of the metal average concentration in the specimen. The blood is then diluted in a simple ratio of 1 part sample, 1 part water, and 48 parts diluent. The diluent contains tetramethylammonium hydroxide (TMAH, 0.4% v/v) and Triton X-100TM (0.05%), which solubilize blood components, and pyridine-2-thiol N-oxide ammonium salt (APDC) (0.01%), aiding in the dissolution of metals released from the biological matrix. Ethanol (1%) facilitates the dissolution of blood components and promotes aerosol generation by reducing the solution’s surface tension. The processed liquid sample is introduced into the mass spectrometer through an inductively coupled plasma (ICP) ionization source. Fine aerosol droplets pass through the plasma region, where thermal energy causes evaporation of the droplets, leading to molecular desolvation of the sample, followed by atomic ionization. Ions first pass through a focusing region, then enter a dynamic reaction cell (DRC), a quadrupole mass filter, and finally undergo selective counting in a detector in a rapid sequence, enabling the determination of various isotopes of elements.

### Measurements of HBV markers

According to the NHANES procedures, the determination of the three biomarkers utilized VITROS reagent kits and calibrators, performed on the VITROS ECi/ECiQ or VITROS 3600 Immunoassay System. Competitive immunoassay technology was employed, with horseradish peroxidase (HRP) as the labeling agent. The levels of antigens or antibodies present in the sample were measured through a chemiluminescent reaction, demonstrating the binding of HRP conjugates.

The final result for the VITROS HBsAg test, expressed in signal-to-cutoff ratio (s/c), is considered negative when < 1.00. A sample with a result > 5.00 is interpreted as positive for HBsAg. Samples with a result ≥ 1.00 and ≤ 5.00 are considered reactive for HBsAg. If the reactivity result is confirmed by supplementary testing, such as with the VITROS Immunoassay Product HBsAg Confirmation Reagent Kit, then the specimen is considered positive for HBsAg. The sample is categorized as “Negative” for HBsAb when the results < 5.00 mIU/mL and as “Positive” for values ≥ 12 mIU/mL. Results falling between ≥ 5.00 mIU/mL and < 12.0 mIU/mL are deemed “Indeterminate”. The initial VITROS HBcAb test result is regarded as positive when the s/c is < 0.90. A specimen is considered negative for HBcAb when the result falls within the range of 1.10 to 4.80. In cases where the result is ≥ 4.8, a retest is conducted after a 1:20 dilution. If the result is ≥ 0.90 but ≤ 1.10, it undergoes two additional confirmatory tests.

### Grouping of HBV markers

Participants were grouped into five categories based on their HBsAg, HBsAb, and HBcAb status: (1) Susceptible: negative for all HBV infection markers; (2) Vaccinated: negative for HBsAg and HBcAb, and positive for HBsAb; (3) Natural Infection: negative for HBsAg, and positive for HBsAb and HBcAb; (4) Acute/Chronic HBV Infection: positive for HBsAg, negative or positive for HBsAb and HBcAb. (5) Others: markers with irregular combinations, 258 persons, this group was not included in the analysis (Table 1).

**Table 1:** Groups of HBV infection

Serological marker	Abbreviation	Susceptible	Vaccinated	Natural Infection	Acute/Chronic HBV infection	Others
Hepatitis B Surface Antigen	HBsAg	-	-	-	+	-
Hepatitis B Surface Antibody	HBsAb	-	+	+	+/-	-
Hepatitis B Core Antibody	HBcAb	-	-	+	+/-	+

### Covariates

All covariates chosen in this research were based on previous studies and clinical experience. The demographic variables were selected as age, gender, body mass index (BMI), race/ethnicity, education level, ratio of family income to poverty (PIR) and marital status. According to the definition of overweight and obesity for adults by WHO, we classified BMI into three categories: < 25 kg/m^2^ (under or normal weight), 25–30 kg/m^2^ (overweight) and > 30 kg/m^2^ (obesity). The race/ethnicity consisted of Mexican American and other Hispanic, Non-Hispanic White and Multi-Racial, Non-Hispanic Black, and Non-Hispanic Asian as described in NHANES protocol. PIR and education level (less than 9th grade, 9-11th grade, high school graduate/GED or equivalent, some college or AA degree, and college graduate or above) were considered to evaluate the socioeconomic status. Marital status was categorized as married or living with partner, divorced or separated, and never married. Three variates including smoke, diabetes, and high blood pressure were defined by the questions separately “Do you now smoke cigarettes”, “Doctor told you have diabetes”, and “Ever told you had high blood pressure” in questionnaire data of NHANES. The following laboratory tests were used to predict liver function: high-density lipoprotein (HDL) /cholesterol, ALP, AST, ALT, AST/ALT.

### Statistical analysis

We combined data from 6 cycles of NHANES (2007–2018) and adjusted the sample weights (MEC exam weights) by dividing them by 6, following the guidance provided in the NHANES tutorials (https://wwwn.cdc.gov/nchs/nhanes/tutorials/default.aspx). This adjustment accounts for the oversampling of subgroups in the dataset. Continuous variables exhibiting a normal distribution were expressed with mean ± standard deviation (Mean ± SD), while skewed variables including metal concentrations were represented with geometric mean or quartiles (Q). Categorical variables were reported as cases (n) and percentage (%). Weighted linear regression and weighted chi-square test were employed to assess the variations among four HBV groups, considering continuous and categorical variables, respectively. Multivariate logistic regression models and binary logistic regression were used to calculate odds ratio (OR) and 95% confidential intervals (CI) for association between metals and HBV infection. A dose-response relationship between heavy metals and biomarkers was illustrated using a generalized additive model by fitting smooth curves. Stratified analyses were also conducted to investigate the potential effect modification of race/ethnicity and BMI on the association between metal exposure and HBV infection. The IBM SPSS Statistics® (v.27 https://www.ibm.com/spss) and EmpowerStats® (v.4.1 http://www.empowerstats.net/) were selected to extract and analysis data. *P* value less than 0.05 were considered statistically significant.

## Results

### Characteristics of participants

Table 2 presented the general characteristics of a total of 8431 participants in the NHANES from 2007 to 2018 with mean age of 46.75 ± 17.46 years. The age of Vaccinated group was 34.52 ± 14.16 years, significantly lower than the other three groups (*P* < 0.0001). The total numbers of men (49.09%, *n* = 4 139) and women (50.91%, *n* = 4 292) were almost same. However, the number of females (56.18%, *n* = 992) with protective antibodies is higher, resulting in a lower infection rate (44.30%, *n* = 385) compared to males (55.70%, *n* = 484). The participants were stratified into three groups based on BMI, comprising 30.70% (*n* = 2 588), 35.31% (*n* = 2 977), and 33.99% (*n* = 2 866) of the total sample size, respectively. Among the Vaccinated and Acute/Chronic Infection, BMI < 25 kg/m^2^ gave a high proportion, 42.34% (*n* = 747) and 48.39% (*n* = 50) respectively. Most of the participants were Non-Hispanic White (68.12%, *n* = 5 743), well-educated (60.92% above college, *n* = 5 136), married or living with partner (65.54%, *n* = 5 526), non-smokers (81.57%, *n* = 6 877), without diabetes (88.67%, *n* = 7 476) and high blood pressure (67.20%, *n* = 5 666) after adjusting the weights. Compared with other three groups, in the Acute/Chronic Infection, the infection rates of Mexican American and Hispanic and Non-Hispanic White and Multi-Racial showed a lower proportion, 7.72% (*n* = 8) and 24.64% (*n* = 15), while Non-Hispanic Asian with a highest proportion, 48.45% (*n* = 50). Participants with the lowest education level, “Less than 9th grade,” were less likely to acquire protective antibodies through vaccination (1.67%, *n* = 29). Similarly, individuals in the Infection showed a lower PIR (2.57 ± 1.56 and 2.47 ± 1.49). In Vaccinated, the proportion of individuals unaffected by diabetes and hypertension was comparatively high, 94.08% (*n* = 1 661) and 81.80% (*n* = 1 444) separately. The testing results of AST (31.30 ± 19.20 U/L) and ALT (35.51 ± 31.92 U/L) in Acute/Chronic Infection were higher than other groups (*P* < 0.05).

**Table 2:** Characteristics of participants from the NHANES 2007–2018

	Total	Susceptible	Vaccinated	Natural Infection	Acute/Chronic HBV Infection	* P* value
N	8 431	5 436	1 765	869	103	
Age, mean ± SD	46.75 ± 17.46	49.65 ± 16.81	34.52 ± 14.16	55.55 ± 14.73	48.45 ± 16.54	< 0.0001
Gender, n (%)						< 0.0001
Male	4 139 (49.09)	2 719 (50.02)	773 (43.82)	484 (55.70)	52 (50.93)	
Female	4 292 (50.91)	2 717 (49.98)	992 (56.18)	385 (44.30)	51 (49.07)	
BMI, n (%)						< 0.0001
< 25	2 588 (30.70)	1 457 (26.80)	747 (42.34)	267 (30.76)	50 (48.39)	
25 ≤ BMI ≤ 30	2 977 (35.31)	2 023 (37.22)	516 (29.26)	311 (35.78)	31 (30.44)	
> 30	2 866 (33.99)	1 956 (35.98)	501 (28.40)	291 (33.47)	22 (21.17)	
Race/Ethnicity, n (%)						< 0.0001
Mexican American and Hispanic	1 234 (14.64)	821 (15.11)	253 (14.36)	104 (11.92)	8 (7.72)	
Non-Hispanic White and Multi-Racial	5 743 (68.12)	3 922 (72.14)	1 185 (67.13)	360 (41.46)	25 (24.64)	
Non-Hispanic Black	948 (11.24)	527 (9.69)	199 (11.29)	198 (22.73)	20 (19.19)	
Non-Hispanic Asian	506 (6.00)	166 (3.06)	127 (7.22)	208 (23.89)	50 (48.45)	
Education level, n (%)						< 0.0001
Less than 9th grade	457 (5.42)	331 (6.08)	29 (1.67)	76 (8.80)	12 (11.32)	
9-12th grade	841 (9.98)	573 (10.54)	125 (7.06)	116 (13.30)	9 (8.49)	
High school graduate /GED	1 715 (20.34)	1 200 (22.08)	252 (14.27)	183 (21.07)	20 (19.67)	
College or AA degree	2 596 (30.79)	1 614 (29.70)	617 (34.98)	245 (28.19)	30 (29.34)	
College graduate or above	2 540 (30.13)	1 566 (28.80)	634 (35.92)	247 (28.37)	28 (27.06)	
Unkown	282 (3.34)	152 (2.80)	109 (6.10)	2 (0.27)	4 (4.12)	
PIR, mean ± SD	2.82 ± 1.64	2.85 ± 1.63	2.87 ± 1.68	2.57 ± 1.56	2.47 ± 1.49	< 0.0001
Marital status, n (%)						< 0.0001
Married or living with partner	5 526 (65.54)	3 747 (68.93)	946 (53.59)	605 (69.61)	67 (65.38)	
Divorced or separated	1 064 (12.62)	749 (13.78)	149 (8.44)	121 (13.91)	14 (13.44)	
Never married	1 559 (18.49)	789 (14.51)	562 (31.85)	140 (16.16)	17 (16.38)	
Unkown	282 (3.35)	151 (2.78)	108 (6.12)	3 (0.32)	5 (4.80)	
Smoking Status, n (%)						< 0.0001
Yes	1 554 (18.43)	1 018 (18.72)	313 (17.71)	153 (17.59)	13 (12.96)	
No	6 877 (81.57)	4 418 (81.28)	1 452 (82.29)	716 (82.41)	90 (87.04)	
Diabetes, n (%)						< 0.0001
Yes	756 (8.97)	543 (9.99)	69 (3.89)	127 (14.59)	10 (9.33)	
No	7 476 (88.67)	4 767 (87.69)	1 661 (94.08)	717 (82.55)	89 (86.41)	
Doubtful	196 (2.32)	124 (2.28)	35 (2.01)	25 (2.86)	4 (4.26)	
Unkown	3 (0.04)	2 (0.04)	-	-	-	
High Blood Pressure, n (%)						< 0.0001
Yes	2 622 (31.10)	1 914 (35.21)	318 (18.03)	276 (31.73)	21 (20.82)	
No	5 666 (67.20)	3 519 (64.73)	1 444 (81.8)	427 (49.09)	67 (64.15)	
Unkown	143 (1.70)	3 (0.06)	3 (0.17)	167 (19.18)	15 (15.03)	
HDL/Cholesterol (mg/dL), mean ± SD	53.06 ± 15.51	52.78 ± 15.68	54.03 ± 14.78	53.23 ± 16.18	54.31 ± 17.65	0.0107
ALP (IU/L), mean ± SD	66.06 ± 23.74	66.71 ± 24.08	62.35 ± 21.65	70.64 ± 25.54	70.20 ± 27.56	< 0.0001
AST (U/L), mean ± SD	25.17 ± 15.16	25.46 ± 13.67	24.34 ± 19.08	24.56 ± 14.61	31.30 ± 19.20	0.0012
ALT (U/L), mean ± SD	24.61 ± 17.26	24.93 ± 17.35	23.82 ± 17.24	23.40 ± 14.60	35.51 ± 31.92	< 0.0001
AST/ALT, mean ± SD	1.13 ± 0.35	1.13 ± 0.35	1.14 ± 0.34	1.15 ± 0.35	1.05 ± 0.32	0.1883

Mean ± SD for continuous variables, *P* value calculated via weighted linear regression. % for categorical variables, *P* value calculated via weighted chi-square test

### Heavy metals concentration differences among HBV groups

Geometric mean of concentrations of five heavy metals were compared. As depicted in Table 3, blood Pb, blood Cd, blood Hg and blood Mn were significantly higher in Natural Infection and Acute/Chronic Infection than others (all *P* < 0.001), while blood Se without difference (*P* = 0.421) (Table 3).

**Table 3:** Geometric Mean of metals in HBV groups

GM (95%CI)	Susceptible	Vaccinated	Natural Infection	Acute/Chronic HBV Infection	* P*-value
Pb (µg/dL)	1.12 (1.10 1.15)	0.82 (0.80 0.85)	1.39 (1.33 1.45)	1.31 (1.18 1.46)	< 0.001
Cd (µg/L)	0.35 (0.34 0.36)	0.30 (0.29 0.31)	0.44 (0.42 0.46)	0.49 (0.42 0.58)	< 0.001
Hg (µg/L)	0.82 (0.80 0.84)	0.90 (0.86 0.95)	1.55 (1.43 1.67)	2.26 (1.82 2.81)	< 0.001
Se (µg/L)	192.98 (192.29 193.67)	193.00 (191.93 194.08)	193.55 (191.69 195.44)	188.36 (183.45 193.41)	0.421
Mn (µg/L)	9.14 (9.05 9.22)	9.71 (9.55 9.87)	9.84 (9.61 10.08)	11.19 (10.44 12.00)	< 0.001

*P* value: Kruskal-Wallis for continuous variables, Fisher’s exact for count variables(if theoretical counts < 10)

### The relationship between heavy metals and HBV groups

A multivariate logistic regression model was used to evaluate the association between heavy metals and HBV groups. As the increase of concentration of blood Pb and blood Cd, the protection of vaccination decreased to 0.29 (95%CI: 0.29–0.29) and 0.62 (95%CI: 0.62–0.62). While with the progressive elevation of blood Pb concentrations, a notable upward trend in the OR is observed among individuals in the Natural Infection (Q2: OR 1.26, 95%CI 1.25–1.26; Q3: OR 1.74, 95%CI 1.73–1.74; Q4: OR 1.87, 95%CI 1.87–1.88), the same trend showed in blood Mn (Q2: OR 1.08, 95%CI 1.08–1.08; Q3: OR 1.18, 95%CI 1.17–1.18; Q4: OR 1.58, 95%CI 1.58–1.59). The ORs among individuals in the Acute/Chronic Infection also exhibits a discernible increasing trend as the concentrations of blood Cd (Q2: OR 1.44, 95%CI 1.43–1.46; Q3: OR 1.89, 95%CI 1.87–1.91; Q4: OR 3.05, 95%CI 3.02–3.08) and blood Hg (Q2: OR 1.57, 95%CI 1.55–1.59; Q3: OR 2.81, 95%CI 2.77–2.84; Q4: OR 6.76, 95%CI 6.68–6.84) (Table 4).

**Table 4:** Multivariate logistic regression model between heavy metals and HBV groups

	Susceptible OR (95%CI)	Vaccinated OR (95%CI)	Natural infection OR (95%CI)	Acute/Chronic HBV infection OR (95%CI)	* P* value
Pb (µg/dL)					
Q1 (0.07 0.67)	ref.	ref.	ref.	ref.	
Q2 (0.67 1.06)	1.23 (1.23, 1.23)	0.51 (0.51, 0.51)	1.26 (1.25, 1.26)	1.96 (1.94, 1.99)	< 0.0001
Q3 (1.06 1.68)	1.14 (1.14, 1.15)	0.32 (0.32, 0.33)	1.74 (1.73, 1.74)	3.03 (3.00, 3.06)	< 0.0001
Q4 (1.68 61.29)	1.08 (1.08, 1.08)	0.29 (0.29, 0.29)	1.87 (1.87, 1.88)	2.67 (2.63, 2.70)	< 0.0001
Cd (µg/L)					
Q1 (0.07 0.19)	ref.	ref.	ref.	ref.	
Q2 (0.19 0.32)	1.03 (1.03, 1.03)	0.79 (0.79, 0.79)	0.97 (0.97, 0.97)	1.44 (1.43, 1.46)	< 0.0001
Q3 (0.32 0.60)	0.89 (0.89, 0.89)	0.60 (0.60, 0.60)	1.73 (1.72, 1.73)	1.89 (1.87, 1.91)	< 0.0001
Q4 (0.60 9.30)	0.81 (0.81, 0.81)	0.62 (0.62, 0.62)	1.70 (1.70, 1.70)	3.05 (3.02, 3.08)	< 0.0001
Hg (µg/L)					
Q1 (0.11 0.42)	ref.	ref.	ref.	ref.	
Q2 (0.42 0.84)	1.09 (1.09, 1.09)	0.81 (0.81, 0.81)	0.98 (0.98, 0.98)	1.57 (1.55, 1.59)	< 0.0001
Q3 (0.84 1.86)	0.99 (0.98, 0.99)	0.90 (0.89, 0.90)	1.23 (1.22, 1.23)	2.81 (2.78, 2.84)	< 0.0001
Q4 (1.86 63.64)	0.77 (0.77, 0.77)	1.04 (1.04, 1.04)	2.27 (2.26, 2.28)	6.76 (6.68, 6.84)	< 0.0001
Se (µg/L)					
Q1 (105.38 178.05)	ref.	ref.	ref.	ref.	
Q2 (178.05 192.55)	1.12 (1.12, 1.12)	0.92 (0.92, 0.92)	0.83 (0.83, 0.84)	0.88 (0.87, 0.88)	< 0.0001
Q3 (192.55 208.39)	1.17 (1.17, 1.17)	0.94 (0.93, 0.94)	0.87 (0.87, 0.87)	0.30 (0.30, 0.30)	< 0.0001
Q4 (208.40 734.80)	1.15 (1.15, 1.15)	0.94 (0.93, 0.94)	0.84 (0.84, 0.84)	0.46 (0.46, 0.47)	< 0.0001
Mn (µg/L)					
Q1 (1.61 7.38)	ref.	ref.	ref.	ref.	
Q2 (7.38 9.25)	1.02 (1.02, 1.02)	1.25 (1.25, 1.25)	1.08 (1.08, 1.08)	2.59 (2.56, 2.62)	< 0.0001
Q3 (9.25 11.70)	0.97 (0.96, 0.97)	1.26 (1.26, 1.27)	1.18 (1.17, 1.18)	2.01 (1.99, 2.04)	< 0.0001
Q4 (11.70 57.77)	0.74 (0.74, 0.74)	1.48 (1.47, 1.48)	1.58 (1.58, 1.59)	5.47 (5.41, 5.53)	< 0.0001

### The relationship between heavy metals and HBsAg, HBcAb and HBsAb

**Fig. 2:**
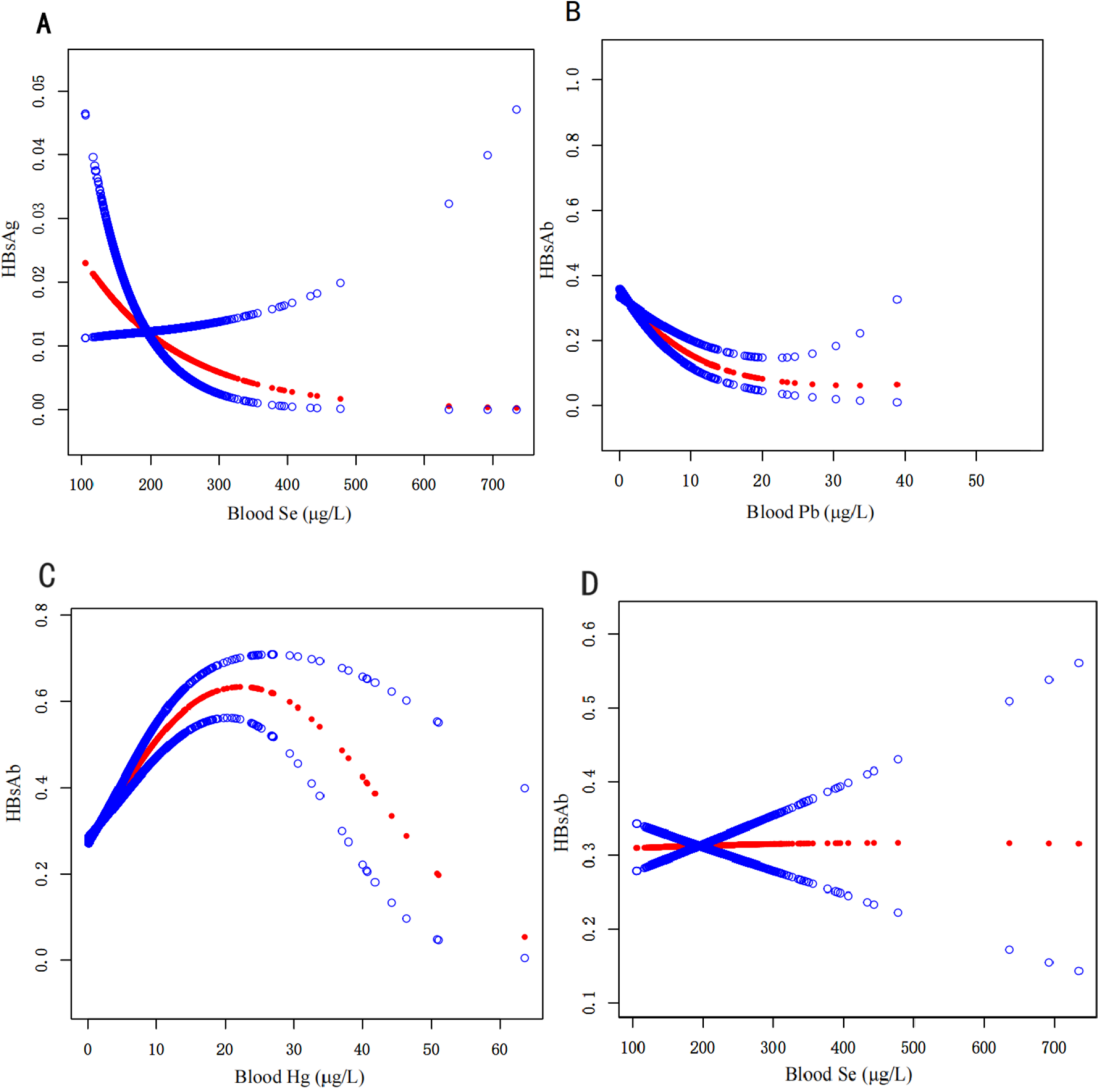
General additive models reveal the correlation between heavy metal concentration and HBV markers

To further elucidate the association between heavy metal exposure and the HBV markers HBsAg, HBcAb and HBsAb, 3 models were constructed to eliminate potential confounding factors, including model 1 (no covariates were adjusted), model 2 (only gender, age, and race/ethnicity), and model 3 (all covariates). Simultaneously, we employed a generalized additive model (depicted in Fig. 2A and D) to intuitively assess the relationship between the concentrations of heavy metals as continuous variables and the hepatitis B infection indicators, HBsAg and HBsAb. A notable risk increase of 1.37-fold and 1.34-fold was observed for the Q2 and Q3 concentrations of blood Pb, respectively, comparing to Q1 in model 3. High concentrations of blood Hg and Mn (Q4) trends to increase the risk of HBV infection by 2.03-fold and 1.52-fold (Table 5). In addition, high concentrations of Hg (Q3: OR 1.30, 95%CI: 1.03–1.65; Q4: OR 1.94, 95%CI 1.54–2.45) and Mn (Q4: OR 1.37, 95%CI 1.09–1.72) can increase the probability of positive HBcAb (Table 6). Elevated levels of Se can mitigate the risk of HBsAg infection (Q3: OR 0.43, 95%CI 0.23–0.79; Q4: OR 0.46, 95%CI 0.26–0.82 and Fig. 2A). While the results in Table 7; Fig. 2B that the high concentration of Pb reduces the protective ability of the HBsAb (Q2: OR 0.84, 95%CI 0.73–0.97; Q3: OR 0.77, 95%CI 0.66–0.90; Q4: OR 0.83, 95%CI 0.70–0.98). Figure 2C reveals that Hg concentrations exceeding 30.58 µg/L also exhibit inhibitory effects on HBsAb. There is no interaction between Se and HBsAb (*P* > 0.05, Table 7), which is further confirmed in Fig. 2D.

**Table 5:** Adjusted associations between five heavy metals and HBsAg

Exposure	Non-adjusted	Adjust I	Adjust II
Pb (µg/dL)			
Q1	ref.	ref.	ref.
Q2	3.25 (1.47, 7.18) 0.0035**	2.40 (1.07, 5.41) 0.0344*	2.37 (1.04, 5.39) 0.0400*
Q3	4.58 (2.13, 9.85) 0.0001**	2.70 (1.20, 6.10) 0.0167*	2.34 (1.01, 5.40) 0.0471*
Q4	3.79 (1.74, 8.27) 0.0008**	2.32 (1.00, 5.41) 0.0506	2.01 (0.84, 4.81) 0.1182
Cd (µg/L)			
Q1	ref.	ref.	ref.
Q2	1.29 (0.64, 2.63) 0.4753	0.92 (0.45, 1.90) 0.8268	0.88 (0.42, 1.83) 0.7325
Q3	2.07 (1.08, 3.98) 0.0292*	1.08 (0.54, 2.15) 0.8342	1.02 (0.49, 2.12) 0.9573
Q4	2.92 (1.56, 5.47) 0.0008**	1.42 (0.72, 2.80) 0.3112	1.32 (0.60, 2.88) 0.4848
Hg (µg/L)			
Q1	ref.	ref.	ref.
Q2	1.75 (0.70, 4.40) 0.2318	1.70 (0.67, 4.29) 0.2601	1.64 (0.65, 4.16) 0.2964
Q3	2.93 (1.24, 6.91) 0.0140*	2.17 (0.92, 5.16) 0.0785	2.24 (0.94, 5.38) 0.0696
Q4	8.81 (4.02, 19.29) < 0.0001**	3.31 (1.45, 7.53) 0.0044**	3.03 (1.31, 7.04) 0.0097**
Se (µg/L)			
Q1	ref.	ref.	ref.
Q2	0.85 (0.52, 1.40) 0.5273	0.77 (0.46, 1.28) 0.3106	0.72 (0.43, 1.22) 0.2254
Q3	0.50 (0.28, 0.89) 0.0189*	0.45 (0.25, 0.82) 0.0085**	0.43 (0.23, 0.79) 0.0068**
Q4	0.67 (0.40, 1.15) 0.1449	0.51 (0.30, 0.89) 0.0172*	0.46 (0.26, 0.82) 0.0078**
Mn (µg/L)			
Q1	ref.	ref.	ref.
Q2	1.74 (0.83, 3.68) 0.1431	1.68 (0.79, 3.60) 0.1802	1.70 (0.79, 3.67) 0.1729
Q3	1.91 (0.92, 3.97) 0.0829	1.45 (0.67, 3.14) 0.3461	1.42 (0.65, 3.11) 0.3781
Q4	4.80 (2.50, 9.23) < 0.0001**	2.68 (1.28, 5.59) 0.0086**	2.52 (1.20, 5.28) 0.0149*

*P < 0.05; **P < 0.01

**Table 6:** Adjusted associations between five heavy metals and HBcAb

Exposure	Non-adjusted	Adjust I	Adjust II
Pb (µg/dL)			
Q1	ref.	ref.	ref.
Q2	2.29 (1.84, 2.85) < 0.0001**	1.24 (0.98, 1.58) 0.0768	1.19 (0.92, 1.54) 0.1860
Q3	3.56 (2.89, 4.39) < 0.0001**	1.31 (1.03, 1.66) 0.0259*	1.18 (0.91, 1.54) 0.2036
Q4	3.76 (3.05, 4.63) < 0.0001**	1.17 (0.92, 1.50) 0.2005	0.97 (0.74, 1.27) 0.8075
Cd (µg/L)			
Q1	ref.	ref.	ref.
Q2	1.52 (1.23, 1.87) 0.0001**	0.99 (0.79, 1.25) 0.9652	0.91 (0.71, 1.17) 0.4555
Q3	2.65 (2.17, 3.23) < 0.0001**	1.24 (1.00, 1.55) 0.0548	1.18 (0.92, 1.51) 0.1821
Q4	3.03 (2.49, 3.69) < 0.0001**	1.40 (1.12, 1.75) 0.0030**	1.26 (0.95, 1.66) 0.1094
Hg (µg/L)			
Q1	ref.	ref.	ref.
Q2	1.24 (1.00, 1.53) 0.0470*	1.11 (0.89, 1.39) 0.3562	1.15 (0.90, 1.46) 0.2641
Q3	1.72 (1.41, 2.10) < 0.0001**	1.22 (0.98, 1.51) 0.0716	1.30 (1.03, 1.65) 0.0275*
Q4	3.95 (3.29, 4.75) < 0.0001**	1.71 (1.39, 2.10) < 0.0001**	1.94 (1.54, 2.45) < 0.0001**
Se (µg/L)			
Q1	ref.	ref.	ref.
Q2	0.96 (0.81, 1.14) 0.6417	1.00 (0.83, 1.21) 0.9731	1.05 (0.86, 1.29) 0.6354
Q3	0.84 (0.70, 0.99) 0.0436*	0.85 (0.71, 1.03) 0.1041	0.94 (0.76, 1.16) 0.5467
Q4	0.94 (0.79, 1.11) 0.4642	0.86 (0.71, 1.03) 0.1072	0.93 (0.76, 1.15) 0.4986
Mn (µg/L)			
Q1	ref.	ref.	ref.
Q2	1.04 (0.87, 1.25) 0.6554	1.19 (0.98, 1.45) 0.0804	1.14 (0.91, 1.41) 0.2489
Q3	1.10 (0.92, 1.32) 0.2956	1.10 (0.90, 1.35) 0.3394	1.11 (0.89, 1.38) 0.3760
Q4	1.64 (1.38, 1.94) < 0.0001**	1.41 (1.14, 1.73) 0.0014**	1.37 (1.09, 1.72) 0.0071**

*P < 0.05; **P < 0.01

**Table 7:** Adjusted associations between five heavy metals and HBsAb

Exposure	Non-adjusted	Adjust I	Adjust II
Pb (µg/dL)			
Q1	ref.	ref.	ref.
Q2	0.72 (0.63, 0.82) < 0.0001**	0.87 (0.75, 1.00) 0.0423*	0.84 (0.73, 0.97) 0.0202*
Q3	0.60 (0.52, 0.68) < 0.0001**	0.78 (0.67, 0.91) 0.0013**	0.77 (0.66, 0.90) 0.0014**
Q4	0.55 (0.48, 0.63) < 0.0001**	0.85 (0.72, 1.00) 0.0463*	0.83 (0.70, 0.98) 0.0311*
Cd (µg/L)			
Q1	ref.	ref.	ref.
Q2	0.90 (0.79, 1.02) 0.1070	0.95 (0.82, 1.09) 0.4341	0.92 (0.80, 1.06) 0.2547
Q3	0.87 (0.76, 1.00) 0.0426*	0.96 (0.83, 1.11) 0.5807	0.94 (0.80, 1.10) 0.4394
Q4	0.92 (0.81, 1.05) 0.2420	0.94 (0.81, 1.09) 0.4146	1.08 (0.89, 1.30) 0.4213
Hg (µg/L)			
Q1	ref.	ref.	ref.
Q2	0.92 (0.80, 1.05) 0.2148	0.97 (0.84, 1.12) 0.6602	0.95 (0.82, 1.10) 0.4689
Q3	1.06 (0.93, 1.21) 0.3819	1.13 (0.98, 1.30) 0.0924	1.05 (0.90, 1.21) 0.5528
Q4	1.66 (1.46, 1.89) < 0.0001**	1.47 (1.27, 1.70) < 0.0001**	1.36 (1.16, 1.59) 0.0001**
Se (µg/L)			
Q1	ref.	ref.	ref.
Q2	1.02 (0.90, 1.16) 0.7403	0.95 (0.82, 1.08) 0.4252	0.95 (0.83, 1.10) 0.5002
Q3	0.95 (0.83, 1.08) 0.4429	0.91 (0.79, 1.04) 0.1669	0.91 (0.79, 1.05) 0.2034
Q4	0.95 (0.84, 1.09) 0.4837	0.89 (0.77, 1.02) 0.1036	0.91 (0.78, 1.05) 0.1862
Mn (µg/L)			
Q1	ref.	ref.	ref.
Q2	1.31 (1.14, 1.50) 0.0001**	1.25 (1.08, 1.44) 0.0024**	1.25 (1.08, 1.45) 0.0028**
Q3	1.32 (1.16, 1.51) < 0.0001**	1.13 (0.97, 1.30) 0.1104	1.17 (1.01, 1.36) 0.0407*
Q4	1.72 (1.50, 1.96) < 0.0001**	1.19 (1.02, 1.38) 0.0264*	1.24 (1.06, 1.45) 0.0076**

*P < 0.05; **P < 0.01

### Subgroup analysis stratified by BMI and race

**Fig. 3:**
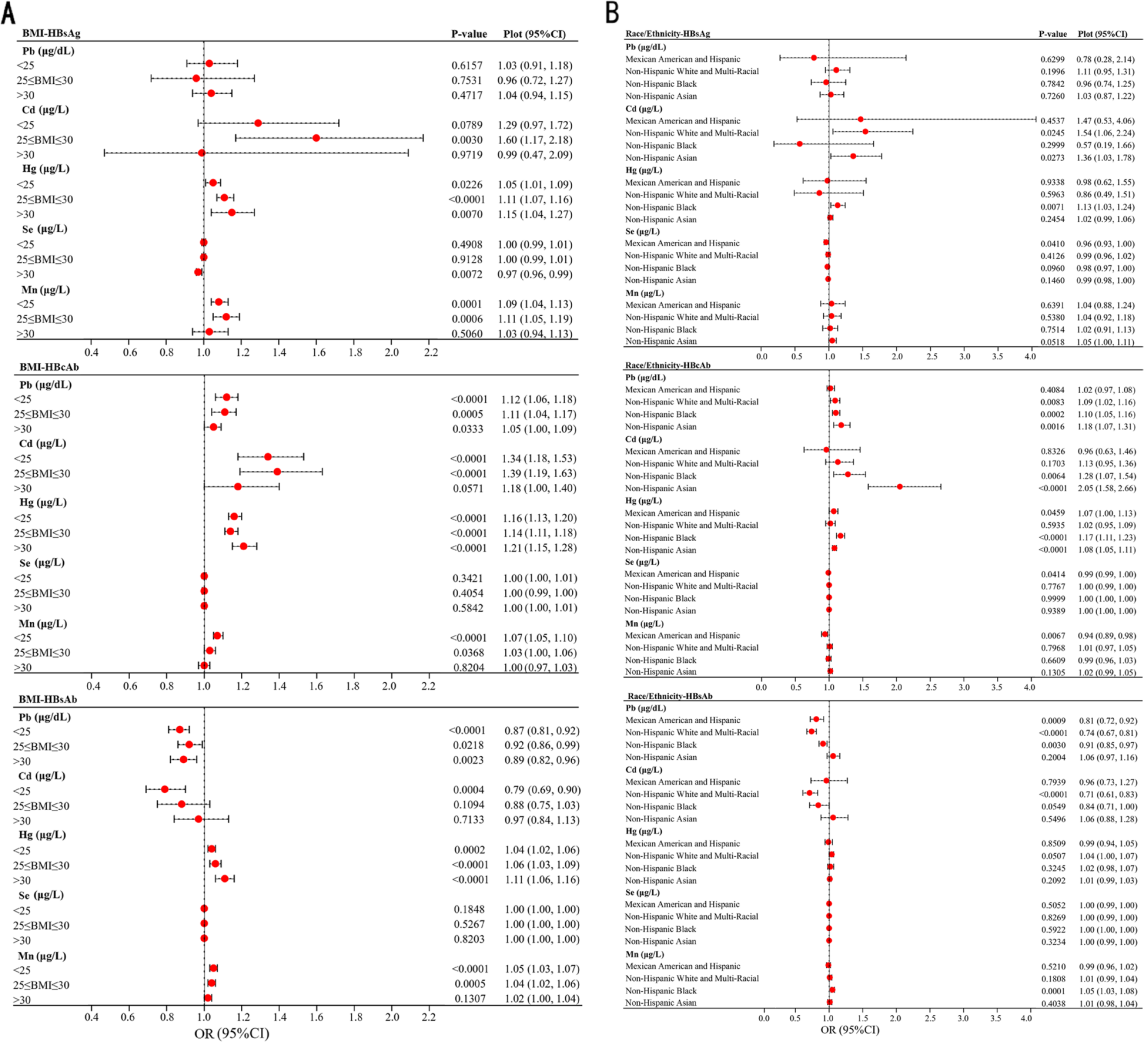
**a** Stratified associations of BMI between heavy metals and HBV markers. **b** Stratified associations of race between heavy metals and HBV

The stratified analyses of potential effect factors of BMI and race/ethnicity were conducted on the association between heavy metals and HBsAg, HBcAb, and HBsAb. The risk of HBsAg infection is positively correlated with blood Hg levels as BMI increases [BMI < 25 kg m^−2^ (OR 1.05, 95%CI 1.01–1.09), ≥ 25 and ≤ 30 kg m^−2^ (OR 1.11, 95%CI 1.07–1.16), > 30 kg m^−2^ (OR 1.15, 95%CI 1.04–1.27)] (Fig. 3a). HBcAb was significantly positively correlated with BMI according to the ORs among blood Pb, blood Cd, and blood Hg in Fig. 3a. Blood Cd increases the risk of HBV infection in Non-Hispanic White (OR 1.54, 95%CI 1.06–2.24) and Non-Hispanic Asian individuals of 36% (OR 1.36, 95%CI 1.03–1.78), while blood Hg elevates the risk in Non-Hispanic Black individuals (OR 1.13, 95%CI 1.03–1.24) (Fig. 3b). Blood Cd increases the probability of positive HBcAb in Non-Hispanic Black individuals (OR 1.28, 95%CI 1.07–1.54) and Non-Hispanic Asian populations (OR 2.05, 95%CI 1.58–2.66). It is noteworthy that Se did not exhibit a significant influence on HBV infection, aligning with the congruent findings from the preceding data analysis.

## Discussion

The rapid progression of industrialization and the significant increase in electronic device production have heightened the susceptibility of individuals to heavy metal exposure, even as they enjoy the convenience and benefits offered by electronic products [27]. This is due to the release of heavy metals into water, soil, and air during the degradation of electronic waste, posing both evident and hidden hazards to human health [28]. Despite a decline in the exposure to certain heavy metals in developed nations over the past few decades, the persistent threat of low-dose exposure remains due to factors such as long-term inhalation of automobile exhaust, combustion of fossil fuels, smoking, exposure to marine pollution, and dietary intake [29]. This threat is attributed to the prolonged half-life, metabolic challenges, and ease of accumulation associated with these metals, thereby presenting a potential risk to public health [31].

In this study, we explored the correlation between five heavy metals and HBV infection, utilizing NHANES data from 2007 to 2018. Through the comparison of mean values, we observed that the concentrations of heavy metals, except for Se, were higher in individuals with acute and chronic hepatitis B and in those who had a history of natural hepatitis B infection compared to those who were uninfected or immune to hepatitis B (Table 3). This observation could be attributed to the immunotoxicity of certain heavy metals, which may impair antibody production and cellular immunity [33]. It is noteworthy that individuals with HBsAb, which are protective against hepatitis B, exhibit lower levels of Pb in their blood. The multiple logistic regression analysis (Table 4) and adjusted models (Table 7) in this study revealed a negative correlation between immunity against HBV and increasing blood Pb concentration, particularly in the highest quartile (1.68 ~ 61.29). For every 1 µg/dL increase in blood Pb levels, the immunity against HBV decreased by 17% (OR 0.83, 95%CI 0.70–0.98) (Table 7), but the risk of infection would increase (Q2: OR 2.37, 95%CI 1.04–5.39; Q3: OR 2.34, 95%CI 1.01–5.30) (Table 5). One specific study also indicates that an increase in Pb concentration is associated with a decrease in the concentration of HBsAb, and high concentration Pb exposure can diminish the immune response to the hepatitis B vaccine. For each 1 µg/dL increase in child blood Pb levels, there was a reduction of 0.4467 s/co in HBsAb titers in both groups [26]. Similar reductions were observed in several other vaccine-specific IgG titers (e.g., diphtheria, pertussis, tetanus, Japanese encephalitis, polio, measles, mumps, and rubella) in Pb-exposed children [36]. Similarly, an elevated blood mercury concentration exceeding 30.58 µg/L is associated with an increased risk of HBV infection. For each 1 µg/dL increase in blood Hg level, the probability of HBsAg will increase 2.03 fold (Table 5) (OR 3.03, 95%CI 1.31–7.04). Consequently, in areas where exposure to heavy metals, particularly Pb and Hg, is prevalent, where there is a combination of decreased antibody effectiveness and heightened infection risk, there is a need for a more targeted approach to vaccination strategies.

After conducting univariate analysis of all covariates, our study found that BMI and ethnicity might influence the relationship between heavy metals and HBV infection. These results align with previous findings reported in the literature [38]. Subsequently, we identified a significantly high prevalence of hepatitis B infection among Non-Hispanic Asians, constituting only 6% of the population, yet demonstrating an infection rate as high as 48.45%. Additionally, a remarkable age disparity was observed, with the vaccinated group (34.52 ± 14.16 years) (Table 2) consisting of significantly younger individuals compared to the other groups. These findings imply that with a gradual of health awareness, people choice to undergo hepatitis B vaccination, resulting in the development of protective antibodies against the virus. Overall, these results underscore the crucial role of hepatitis B vaccination in safeguarding public health.

To the best of our knowledge, this study is the first to explore the differences in blood heavy metal concentrations among various groups by categorizing hepatitis B markers, aiming to elucidate the relationship between heavy metals and hepatitis B immunity, as well as the risk of hepatitis B infection. However, our study has several limitations that need to be acknowledged. Firstly, we excluded 258 individuals from the study, constituting the “Others” group, which including those only HBcAb positive and HBsAg, HBsAb negative. The exclusion of this population group has two main reasons: (1) HBcAb, as a marker of HBV, with a great sensitivity,which would lead to a higher chance of producing false positives [40]; (2) Only using HBcAb as the single biomarker to screen donated blood for occult HBV infection is not recommended, even in resource-limited settings, because assessing latent hepatitis B infection solely on the basis of HBcAb positivity is still very controversia [42]. Secondly, since this is a cross-sectional study relying on NHANES data, where all populations are sampled and surveyed at a single point in time with no follow-up or tracing, and lacking comprehensive data on metal metabolism, such as urinary concentrations of specific metal species. Additionally, the tests for hepatitis B markers in this data set are limited to negative-positive results, without providing specific titers. Consequently, the exact relationship between blood heavy metal concentrations and hepatitis B marker titers cannot be fully elucidated in this paper. Thirdly, it is important to acknowledge that the NHANES database primarily represents the American population, which largely represents of developed countries with a declining incidence of hepatitis B in recent decades. Therefore, the number of participants with acute and chronic hepatitis B infections in our study is relatively small, comprising only 103 individuals. To strengthen the validity and extend the applicability of our findings, we have planned future studies to investigate the association between blood heavy metal concentrations and the risk of hepatitis B infection and immunity through measuring the concentrations of heavy metals in blood and metabolized species several times in the Chinese population, where hepatitis B prevalence is notably higher. By conducting these subsequent studies, we aim to provide a more comprehensive understanding of the relationship between heavy metals and hepatitis B, thereby enhancing the significance and depth of our research.

## Conclusion

In summary, our findings indicate a reduction in blood Se concentration and an elevation in blood concentrations of Hg and Mn among the HBsAg-positive population. No statistically significant correlation was observed between Se and the other two hepatitis B markers. Additionally, our investigation revealed that increased Pb content diminishes the protective efficacy of HBsAb, rendering vaccine failure more likely. These outcomes imply a connection between blood heavy metal levels and the risk of HBV infection, prompting consideration for reevaluating HBV immunization strategies.

## Data Availability

The datasets generated and/or analysed during the current study are available in the OneDrive repository, rawdata.xls.

## Abbreviations

ALP: Alkaline phosphatase
ALT: Alanine transaminase
AST: Aspartate transaminase
BMI: Body mass index
Cd: Cadmium
CHB: Chronic hepatitis B
CI: Confidential intervals
GGT: Gamma-glutamyl transferase
HBV: Hepatitis B virus
HBcAb: Hepatitis B core antibodies
HBcAg: HBV core antigen
HBsAg: HBV surface antigen
HBsAb: Hepatitis B surface antibodies
HCC: Hepatocellular carcinoma
HD: High-density lipoprotein
Hg: Mercury
Mn: Manganese
NAFLD: Non-alcoholic fatty liver disease NAFLD
NHANES: National Health and Nutrition Examination Survey database
OR: Odds ratio
Pb: Lead
Se: Selenium
